# Stretch-induced increase in cardiac contractility is independent of myocyte Ca^2+^ while block of stretch channels by streptomycin improves contractility after ischemic stunning

**DOI:** 10.14814/phy2.12486

**Published:** 2015-08-19

**Authors:** Samhita S Rhodes, Amadou K S Camara, Mohammed Aldakkak, James S Heisner, David F Stowe

**Affiliations:** 1 Department of Anesthesiology, Medical College of WisconsinMilwaukee, Wisconsin, USA; 2 School of Engineering, Grand Valley State UniversityGrand Rapids, Michigan, USA; 3 Cardiovascular Research Center, Medical College of WisconsinMilwaukee, Wisconsin, USA; 4 Department of Physiology, Medical College of WisconsinMilwaukee, Wisconsin, USA; 5 Research Service, Zablocki VA Medical CenterMilwaukee, Wisconsin, USA; 6 Department of Biomedical Engineering, Marquette UniversityMilwaukee, Wisconsin, USA

**Keywords:** Contractility, cross-bridges, heart, mathematical modeling, myoplasmic calcium, stretch channels

## Abstract

Stretching the cardiac left ventricle (LV) enhances contractility but its effect on myoplasmic [Ca^2+^] is controversial. We measured LV pressure (LVP) and [Ca^2+^] as a function of intra-LV stretch in guinea pig intact hearts before and after 15 min global stunning ± perfusion with streptomycin (STM), a stretch-activated channel blocker. LV wall [Ca^2+^] was measured by indo-1 fluorescence and LVP by a saline-filled latex balloon inflated in 50 μL steps to stretch the LV. We implemented a mathematical model to interpret cross-bridge dynamics and myofilament Ca^2+^ responsiveness from the instantaneous relationship between [Ca^2+^] and LVP ± stretching. We found that: (1) stretch enhanced LVP but not [Ca^2+^] before and after stunning in either control (CON) and STM groups, (2) after stunning [Ca^2+^] increased in both groups although higher in STM versus CON (56% vs. 39%), (3) STM-enhanced LVP after stunning compared to CON (98% vs. 76% of prestunning values), and (4) stretch-induced effects on LVP were independent of [Ca^2+^] before or after stunning in both groups. Mathematical modeling suggested: (1) cooperativity in cross-bridge kinetics and myofilament Ca^2+^ handling is reduced after stunning in the unstretched heart, (2) stunning results in depressed myofilament Ca^2+^ sensitivity in the presence of attached cross-bridges regardless of stretch, and (3) the initial mechanism responsible for increased contractility during stretch may be enhanced formation of cross-bridges. Thus stretch-induced enhancement of contractility is not due to increased [Ca^2+^], whereas enhanced contractility after stunning in STM versus CON hearts results from improved Ca^2+^ handling and/or enhanced actinomyosin cross-bridge cycling.

## Introduction

Myoplasmic calcium ([Ca^2+^]) is the preeminent regulator of myocardial contractility via three major mechanisms. These are (1) the upstream mechanism that is related to the gross concentration of [Ca^2+^], (2) the central mechanism that is related to the sensitivity of the regulatory protein troponin C (TnC) to the available [Ca^2+^], and lastly, (3) the downstream mechanism that is related to the interaction between contractile proteins actin and myosin, and actinomyosin cross-bridge cycling. Myocardial stretch improves contractile function acutely by the Frank-Starling mechanism and more slowly due to the slow force response (von Lewinski et al. 2003). Although stretch-induced improvement in contractile function has been linked to increased Na^+^ and K^+^ flux (Bustamante et al. 1991; Calaghan and White 1999; Ward et al. 2008), the acute effect of stretch on enhancing contractility because of enhancing intracellular [Ca^2+^] is unclear (Kentish and Wrzosek 1998; Calaghan and White 1999; Calaghan et al. 2003; Yeung et al. 2005; Sukharev and Sachs 2012).

We hypothesized that myocardial stretch would result in an acute increase in [Ca^2+^] via stretch-activated cation channels to improve contractility via the upstream mechanism. We sought to elucidate the contribution of these mechanosensitive channels to the phase–space relationship between simultaneously obtained phasic LVP and [Ca^2+^] in stretched hearts before and after short ischemic stunning. To do so we examined several interrelationships of beat-to-beat phasic LVP and [Ca^2+^] in the absence and presence of streptomycin (STM), a known blocker of sarcolemmal stretch-activated cation channels (Yeung et al. 2005). Moreover, we examined these interrelationships after a brief period of ischemia (stunning) to assess if stunning might be mediated in part by stretch activated-cation channels as assessed by blocking stretch channels with STM before stunning.

We also implemented a mathematical model to interpret cross-bridge dynamics and myofilament Ca^2+^ responsiveness from the instantaneous relationship between [Ca^2+^] and LVP (Rhodes et al. 2003a,b, 2006a,b) to elucidate the mechanisms responsible for stretch-induced alterations in intracellular Ca^2+^ handling before and after ischemic stunning. We hypothesized that the myocardial stretch would also involve the central and downstream mechanisms to improve contractile function and increase LVP. We also postulated that these stretch-induced alterations in the central and downstream mechanisms might be altered during and after ischemic stunning. Unlike prolonged global ischemia and reperfusion (IR), which can irreversibly damage the heart, short-term ischemia, that is, stunning, and reperfusion is reversible with no permanent tissue damage.

## Materials and methods

### Isolated heart preparation

The investigation conformed to the *Guide for the Care and Use of Laboratory Animals* from the National Institutes of Health (NIH No. 85-23, Revised 1996). Prior approval was obtained from the Medical College of Wisconsin Institutional Animal Care and Use (IACUC) Committee. We have described the preparation of guinea pig hearts for Ca^2+^ measurements previously (Stowe et al. 2000; An et al. 2001; Varadarajan et al. 2001; Chen et al. 2002; Rhodes et al. 2003a). Ketamine (10 mg/kg) and heparin (5 mL/kg of 1000 U/mL) were injected intraperitoneally into albino English short-haired guinea pigs 15 min before they were decapitated when unresponsive to noxious stimulation. After thoracotomy, the inferior and superior venae cavae were ligated and the aorta was cannulated distal to the aortic valve. Each heart was immediately perfused via the aortic root with a cold oxygenated, modified KR solution (equilibrated with 97% O_2_ and 3% CO_2_) at an aortic root perfusion pressure of 55 mmHg and was then rapidly excised. The KR perfusate (pH 7.4 ± 0.01, pO_2_ 560 ± 10 mmHg) was filtered (5 μm pore size) in-line and had the following calculated composition in mmol/L (nonionized): Na^+^ 138, K^+^ 4.5, Mg^2+^ 1.2, Ca^2+^ 2.5, Cl^−^ 134, HCO_3_^−^ 15, H_2_PO_4_^−^ 1.2, glucose 11.5, pyruvate 2, mannitol 16, EDTA 0.05, probenecid 0.1, and insulin 5 (U/L). Perfusate and bath temperatures were maintained at 37.2 ± 0.1°C using a thermostatically controlled water circulator.

Left ventricular pressure (LVP) was measured isovolumetrically with a transducer connected to a thin and compliant saline-filled latex balloon inserted into the left ventricle (LV) through the mitral valve from a cut in the left atrium. Balloon volume was initially adjusted to a diastolic LVP of zero mmHg so that any subsequent increase in diastolic LVP reflected an increase in LV wall stiffness or diastolic contracture. Saline was added in 50 μL increments to a maximum of 200 μL to stretch the LV. At each volume level the heart was allowed to stabilize for 2 min. Saline was removed in the same way to return the LV volume to baseline values after each volume increase. Pairs of bipolar electrodes were placed in the right atrial appendage, right ventricular apex, and LV base to monitor spontaneous heart rate (HR). Coronary flow (aortic inflow, CF) was measured at a constant perfusion pressure (55 mmHg) by an ultrasonic flowmeter (Transonic T106X, Ithaca, NY) placed directly into the aortic inflow line.

### Measurement of cytosolic free Ca^2+^ in intact hearts

Experiments were carried out in a light-blocking Faraday cage. The heart was partially immobilized by hanging it from the aortic cannula, the pulmonary artery catheter, and the LV balloon catheter. The heart was immersed continuously in the bath at 37°C. The distal end of a trifurcated fiber silica fiberoptic cable (optical surface area 3.85 mm^2^) was placed gently against the LV epicardial surface through a hole in the bath. A rubber O ring was placed over the fiberoptic tip to seal the hole and netting was applied around the heart for optimal contact with the fiber optic tip. This maneuver did not affect LVP. Background auto fluorescence was determined for each heart after initial perfusion and equilibration at 37°C.

Myoplasmic [Ca^2+^] was measured using indo-1 as initially described (Grynkiewicz et al. 1985) and applied to isolated heart preparations (Brandes et al. 1993a,b; Rhodes et al. 2006a; Aldakkak et al. 2011). Indo-1 AM (Sigma Chemical, St. Louis, MO) was freshly dissolved in 1 mL of dimethyl sulfoxide (DMSO) containing 16% (w/v) Pluronic I-127 (Sigma Chemical) and diluted to 165 mL with modified KR solution. Each heart was then loaded with indo-1 AM for 20–30 min with the re-circulated KR solution at a final indo-1 AM concentration of 6 μmol/L. Loading was stopped when the fluorescence (F) intensity at 405 nm increased by about 10-fold. Residual interstitial indo-1 AM was washed out by perfusing the heart with standard perfusate for at least another 20 min. Probenecid (100 μmol/L) was present in the perfusate to retard cell leakage of indo-1. The concentration of DMSO (0.1%) alone had no effect on cardiac performance. We have reported that loading and washout of indo-1 reduces LVP approximately 20%; this effect is due to the negative inotropic effects of the vehicle and mild myoplasmic Ca^2+^ buffering by indo-1 per se (Stowe et al. 2000).

Fluorescence emissions at 405 and 460 nm (F_405_ and F_460_) were recorded using a modified luminescence spectrophotometer (SLM Aminco-Bowman II, Spectronic Instruments, Urbana, IL). The LV region of the heart was excited with light from a xenon arc lamp and the light filtered through a 350 nm monochromator with a bandwidth of 16 nm. The beam was focused onto the in-going fibers of the optic bundle. The arc lamp shutter was opened only for 2.5 sec recording intervals to prevent photobleaching. Emission fluorescence was collected by fibers of the remaining two limbs of the cable and filtered by square interference filters (Corion, Franklin, MA) at 405 and 460 nm. Although both F_405_ and F_460_ decline over time, time control studies showed that the F_405_/F_460_ ratio remains stable indicating no change in effective measured myoplasmic [Ca^2+^] during the course of these studies. The F_405_/F_460_ ratio was converted to [Ca^2+^] after correcting to account for the nonmyoplasmic fraction as described previously (Stowe et al. 2000; An et al. 2001; Varadarajan et al. 2001; Rhodes et al. 2003a) using the following equation (Grynkiewicz et al. 1985):

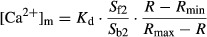
*K*_d_ is 330 nmol/L, *S*_f2_ is the signal intensity of free indo-1 measured at 460 nm and *S*_b2_ is the signal intensity of Ca^2+^-saturated indo-1 measured at 460 nm. Their values were obtained from the *R*_max_ and *R*_min_ experiments, which were done for each preparation. A Ca^2+^ indicator must bind Ca^2+^ to produce a fluorescent transient signal. Thus, the indicator buffer, to some extent, itself binds to the free Ca^2+^ it is intended to measure (Grynkiewicz et al. 1985; Brandes et al. 1993a). The measured whole-cell Ca^2+^ transients are a spatial and temporal summation of local Ca^2+^ transients (Ca^2+^ sparks) that arise from single or clusters of sarcoplasmic reticular Ca^2+^ release channels at the T-tubule-SR microdomain due to influx of Ca^2+^ via L-type Ca^2+^ channels (Rice et al. 1999a).

### Experimental protocol and data collection

**Figure 1:**
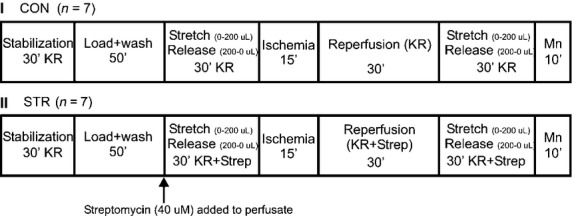
Experimental protocols. Hearts were randomly assigned to either the control IR group (CON) or to the streptomycin-(Strep) treated group IR group (STM). For each group baseline stabilization was followed by loading and wash out of indo-1. Next a stretch and release phase was implemented by adding and removing saline from the latex balloon in increments of 50 to a maximum of 200 μL while measuring phasic LVP with a 2–3 min period of stabilization at each step. Then hearts were subjected to 15 min global ischemia and 30 min reperfusion. This was followed by a repeat of the same stretch and release phase conducted before ischemia. Finally, 10 μmol/L MnCl_2_ was perfusion to quench cytosolic Ca^2+^. In the experimental groups 40 μmol/L streptomycin (STM) was perfused with the KR solution after wash out of indo-1 and STM was continued throughout heart perfusion.

Initial background data were obtained after 30 min stabilization of the heart (Fig.1). After dye loading and wash out, spontaneously beating hearts (approximately 240 beats/min) were randomly assigned for perfusion with STM (40 μmol/L) in KR solution (STM, *n* = 7) or with KR solution alone (CON, *n* = 7) for the remainder of the experimental protocol. Saline was added to, and removed, from the balloon in the LV in steps of 50 μL to a maximal added volume of 200 μL to stretch and release the LV muscle, respectively; at each volume level the heart was allowed to stabilize for 2 min. Hearts were then subjected to 15 min global ischemia followed by 30 min reperfusion ± STM and then restretched and relaxed again as described before. Finally, 100 μmol/L MnCl_2_ was infused for 10 min to quench the myoplasmic indo-1 Ca^2+^ transients so that phasic Ca^2+^ disappeared. This allowed for correction of any nonmyoplasmic contribution to the measured Ca^2+^ transients as described earlier (Varadarajan et al. 2001). All analog signals were digitized (PowerLab®/8 SP; ADInstruments, Castle, Hills, Australia) and recorded at 125 Hz (Chart & Scope v3.63; ADInstruments) on Power Macintosh® G4 computers (Apple, Cupertino, CA) for later analysis using MATLAB® (Mathworks, Natick, MA) and Microsoft Excel® (Microsoft Corporation, Redmond, WA) software. LVP and fluorescence data were digitally low-pass filtered using a 4th order bidirectional Butterworth filter at 20 Hz. After filtering, the F_405_/F_460_ ratio was converted to [Ca^2+^] as detailed previously (Stowe et al. 2000; Varadarajan et al. 2001; Rhodes et al. 2003a). Ca^2+^ transients were corrected to account for the nonmyoplasmic fraction; only myoplasmic [Ca^2+^] data are shown. Developed LVP and myoplasmic [Ca^2+^] after indo-1 washout were not significantly altered over the 185 min experimental period in nonstunned hearts (data not shown).

**Figure 2:**
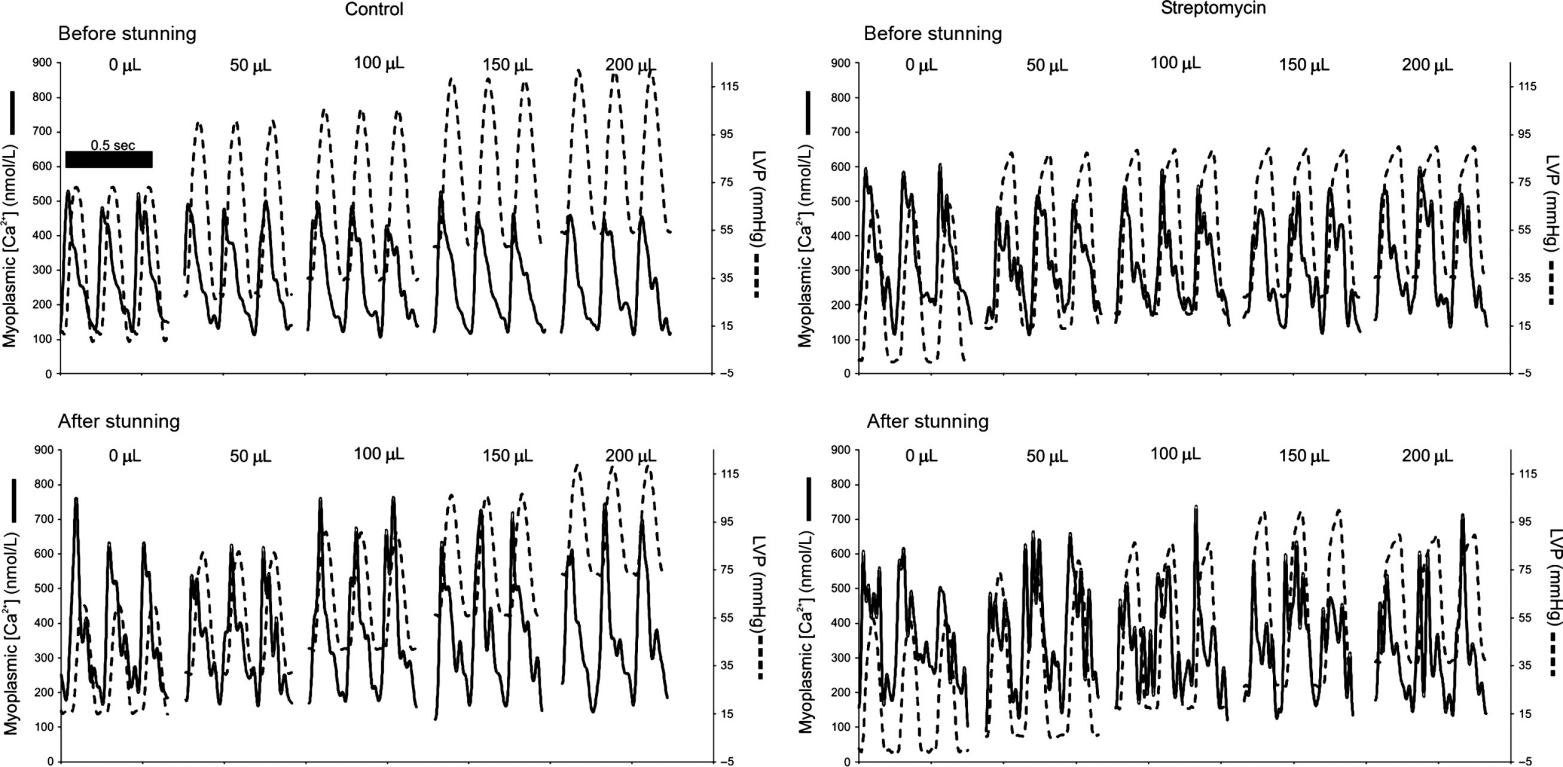
Representative tracings of beat-to-beat phasic [Ca^2+^] (left axis) and LVP (right axis) for three cycles at each stage of myocardial stretch before (top) and after (bottom) stunning for CON and STM groups. As stretch was implemented before stunning in the CON group diastolic and systolic LVP incrementally rose but phasic [Ca^2+^] was unchanged. Compared to the CON group, locking the mechanosensitive cation channels with STM before stunning reduced the absolute values of systolic and diastolic LVP before and after stunning but caused no change in phasic [Ca^2+^]. Note that stunning resulted in increased values of phasic [Ca^2+^] and a higher diastolic LVP but that there were no significant changes in phasic [Ca^2+^] with stretch after stunning between the CON and STM groups.

Simultaneous recordings of [Ca^2+^] and LVP were obtained at selected intervals before and during the stretch protocol and stunning (Fig.2). The timing of peak diastolic [Ca^2+^] for each cardiac cycle was obtained over 2.5 sec recordings using a simple event detection algorithm. The [Ca^2+^] and LVP signals were aligned between each consecutively detected Ca^2+^ diastolic point and averaged on a point-by-point basis to form averaged [Ca^2+^] and LVP transient signals (Rhodes et al. 2003a,b, 2006a,b). LVP was plotted as a function of [Ca^2+^] over a representative cardiac cycle to create phase–space diagrams (Fig.4). These diagrams represent the dynamic relationship between trigger Ca^2+^ and the resulting pressure development due to central and downstream mechanisms and are quantified using LVP· [Ca^2+^] loop area and loop orientation (systolic − diastolic LVP/systolic − diastolic [Ca^2+^]). Because we could not directly measure interactions between Ca^2+^ and troponin C, or kinetics of cross-bridge cycling, we used a previously developed mathematical model to help elucidate effects of changes in myofilament interaction that contribute to changing the dynamic relationship between LVP and Ca^2+^ (Rhodes et al. 2003a,b, 2006a,b).

The experimental protocols for each group, CON and STM, are shown in Figure1. Guinea pig hearts were randomly assigned to either group. Experimental protocols were the same with the exception of adding STM into the perfusate in the one group 30 min before stunning.

### Mathematical model of excitation-contraction coupling

A mathematical model was utilized for the interpretation of the kinetic data. This model allows for the interaction between troponin C attached to the actin (A) myofilament (TnCA) and myosin (M) for cross-bridge formation and force development in the presence of Ca^2+^ (Burkhoff 1994; Baran et al. 1997; Campbell 1997; Shimizu et al. 2002; Rhodes et al. 2003a, 2006a). The model (see Fig.7 and Tables1, 2) consists of four stages governed by the following five differential equations:

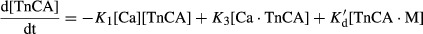


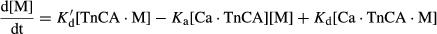


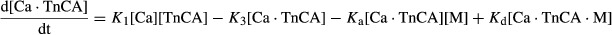


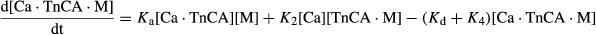


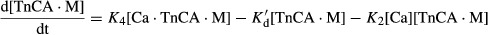


**Table 1:** Model parameters and brief description

Parameter name	Description
*K*_1_ (1/μmol/L·s)	Cooperative binding rate constant of Ca^2+^ to TnCA
*K*_a_ (1/μmol/L·s)	Cooperative rate constant of formation of A·M
*K*_2_ (1/μmol/L·s)	Association rate constant of Ca^2+^ to A·M
*K*_3_ (1/s)	Dissociation rate constant of Ca^2+^ from TnCA
*K*_4_ (1/s)	Dissociation rate constant of Ca^2+^ from A·M
*K*_d_ (1/s)	Dissociation rate constant of A·M in the presence of attached Ca^2+^
*K*_d_′ (1/s)	Dissociation rate constant of A·M in the absence of attached Ca^2+^

*K* = rate constants characterizing the 4-state model are pictured in Figure7 and described in Rhodes et al. (2006a). A = Actin, M = Myosin, TnCA = Troponin C on the A filament, A·M = actinomyosin cross-bridges.

**Table 2:** Effects of stretch before and after stunning on model parameters in CON hearts before and after stunning

Parameter	BL/0 μL	50 μL	100 μL	150 μL	200 μL
Before stunning					
*α*_1_ (1/μmol/L·s)	4.2 ± 0.3	1.8 ± 0.5*	3.0 ± 0.9	2.0 ± 0.7*	3.3 ± 0.5
*α*_a_ (1/μmol/L·s)	6.8 ± 0.6	12.0 ± 1.4*	9.4 ± 0.8*	9.3 ± 1.2*	10.2 ± 1.8*
*β*_1_ (1/μmol/L·s)	1.4 ± 0.5	3.7 ± 1.1	3.3 ± 1.8	6.3 ± 1.8*	1.5 ± 1.0
*β*_a_ (1/μmol/L·s)	118.6 ± 14.5	60.4 ± 17.9*	94.6 ± 27.4	50.9 ± 17.0*	107.5 ± 25.8
*K*_2_ (1/μmol/L·s)	395.2 ± 5.5	367.6 ± 28.9	386.8 ± 13.5	397.5 ± 2.8	401.8 ± 1.0
*K*_3_ (1/s)	481.8 ± 28.1	380.4 ± 33.4*	440.2 ± 10.4	417.2 ± 41.6	431.7 ± 11.5
*K*_4_ (1/s)	16.4 ± 4.4	22.3 ± 3.1	22.0 ± 2.9	17.7 ± 4.1	19.8 ± 2.3
*K*_d_ (1/s)	485.0 ± 23.5	400.2 ± 27.1*	471.4 ± 37.9	450.4 ± 36.3	431.0 ± 14.2
*K*_d_′ (1/s)	649.6 ± 4.3	612.1 ± 36.4	655.7 ± 10.0	647.1 ± 1.5	644.5 ± 0.7
After stunning					
*α*_1_ (1/μmol/L·s)	0.8 ± 0.4#	0.6 ± 0.3#	0.8 ± 0.4#	2.1 ± 1.0*	0.5 ± 0.3#
*α*_a_ (1/μmol/L·s)	3.2 ± 0.4#	18.7 ± 4.3#*	18.3 ± 4.4#*	11.0 ± 2.3*	15.8 ± 3.6*
*β*_1_ (1/μmol/L·s)	11.5 ± 1.0#	6.5 ± 3.2*	6.0 ± 2.6*	5.2 ± 2.9*	5.9 ± 2.4*
*β*_a_ (1/μmol/L·s)	9.4 ± 1.3#	10.4 ± 3.8#	16.1 ± 3.7#*	21.5 ± 6.7#*	13.2 ± 2.6#
*K*_2_ (1/μmol/L·s)	188.1 ± 31.5#	149.7 ± 22.7#	159.4 ± 11.1#	143.3 ± 20.1#*	155.8 ± 2.2#
*K*_3_ (1/s)	322.4 ± 36.6#	194.1 ± 37.5#*	248.7 ± 21.3#*	275.8 ± 48.2#	275.8 ± 6.6#
*K*_4_ (1/s)	35.3 ± 7.5#	34.0 ± 7.4	40.3 ± 8.5#	36.8 ± 9.5#	30.3 ± 7.0
*K*_d_ (1/s)	334.2 ± 44.8#	427.1 ± 84.1	343.9 ± 29.2#	259.9 ± 45.7#*	305.9 ± 38.6#
*K*_d_′ (1/s)	436.1 ± 33.8#	486.0 ± 58.3	407.4 ± 31.3#	336.9 ± 45.4#*	377.8 ± 1.0#

Values are expressed as mean ± SE.

Statistical significance was measured at

* *P* < 0.05 versus BL before or after stunning

# *P *<* *0.05 after versus before ischemia.

LVP, as predicted by the model (LVP_Mod_), is proportional to the number of cross-bridges formed: [Ca·TnCA·M] + [TnCA·M] (Baran et al. 1997). Cooperativity, the positive feedback mechanism responsible for a rise in force, has been attributed to effects of cross-bridge formation on neighboring cross-bridges and/or effects of Ca^2+^ binding to TnCA on neighboring tropomyosin units (Peterson et al. 1991; Rice et al. 1999a,b). In accordance with Baran et al. (1997) and Shimizu et al. (2002), we accounted for cooperative contraction and relaxation by allowing *K*_1_ and *K*_a_ to vary according to functions described as:

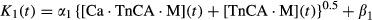


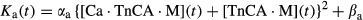


The *α* parameter represents the slopes of the *K*_1_ and *K*_a_ curves and is a measure of sensitivity of the cooperative mechanism; an increase in *α* represents accelerating interactions between TnCA and Ca^2+^ (*α*_1_) and actinomyosin cross-bridges (*α*_a_). The *β* parameter represents the static value of *K*_1_ and *K*_a_ at 0 LVP; an increase in *β* indicates an increase in the resting value of the affinity of TnCA and Ca^2+^ (*β*_1_) and cross-bridge attachment (*β*_a_).

We assumed a “loose coupling” model where Ca^2+^ dissociates from TnCA before the M head detaches from the A molecule (Landesberg and Sideman 1994). We also assumed that the transition between weak and strong cross-bridge conformations is rapid and not rate-limiting (Eisenberg and Hill 1985). Finally, we assumed that changes in sarcomere length have little effect on the relationship between myoplasmic [Ca^2+^] and isovolumic LVP. Kentish and Wrzosek (1998), reported that lengthening of rat isolated myocardium increased twitch force but had no effect on the magnitude of the Ca^2+^ transient, suggesting an increase in myofilament Ca^2+^ sensitivity. In contrast, Shimizu et al. (2002) reported there were no length-dependent alterations in myofilament Ca^2+^ binding or cross-bridge cycling in canine isolated, blood-perfused hearts.

The experimentally measured and averaged Ca^2+^ transients were low-pass filtered at 25 Hz and upsampled to 2500 Hz using a linear interpolation scheme prior to their use as model forcing functions. The governing differential equations were solved numerically using a 4th order Runge-Kutta algorithm (on a MATLAB® platform) with a 0.4 msec step size equal to the postinterpolation sampling interval and the following initial conditions (Peterson et al. 1991; Burkhoff 1994; Baran et al. 1997; Shimizu et al. 2002): [TnCA]_(*t*=0)_ = 70 μmol/L, [M]_(*t*=0)_ = 20 μmol/L, [Ca·TnCA]_(*t*=0)_ = 0 μmol/L, [Ca·TnCA·M]_(*t*=0)_ = 0 μmol/L, and [TnCA·M]_(*t*=0)_ = 0 μmol/L.

Model rate constants were optimized using commercially available algorithms based on constrained quasi-Newton methods that guarantee linear convergence, and were estimated to minimize the root mean square (RMS) error between LVP_Mod_ and measured LVP at the sampled time points.

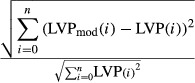


Lower and upper bounds for optimization of 1st order rate constants were set at 0 and 2000 sec (0.5 msec step size) respectively. Initial values for the parameters were obtained from Baran et al. (1997). Several constraints were imposed on the model rate constants during optimization. To ensure a positive feedback, *α*_1_ and *α*_a_ must be greater than 0. *K*_d_′ must be greater than *K*_d_ since cross-bridges dissociate more readily in the absence of attached Ca^2+^; and *K*_d_′ accounts for the physiological difference between contraction and relaxation kinetics (Baran et al. 1997). In addition, the maximum rate constant of Ca^2+^ binding to TnCA with attached cross-bridges must be greater than the maximum rate constant of Ca^2+^ binding to TnCA with no attached cross-bridges (*K*_2_ > *K*_1_); this concept incorporates the idea of a positive feedback mechanism to explain the delay in rise in LVP during contraction (Hill 1983).

### Statistical analysis

Functional indices and model rate constants, computed before and during the stretch protocol and stunning, were compared using one-way ANOVA followed by Dunnett’s comparison of means post hoc test (MINITAB™ Statistical Software Release 13.3; Minitab Inc, State College, PA). Poststunning values from STM and CON groups were compared to their respective prestunning values using Student’s paired *t*-test. Differences among means were considered statistically significant at *P *<* *0.05 (two-tailed). All experimental measurements and model rate constants were expressed as means ± SE.

## Results

### Effects of stretch on contractile function and myoplasmic [Ca^2+^]

Representative tracings of simultaneously obtained LVP and [Ca^2+^] from hearts in the CON and STM groups before and after stunning (Fig.2) showed that stretching the heart before or after stunning by increasing the volume of the balloon caused diastolic LVP to increase in steps. As volume was reduced in the balloon diastolic LVP returned to its prestretched value (data not shown). This effect was observed to a greater extent in the CON group than in the STM group. In both CON and STM groups note that while contractile function was noticeably enhanced with stretch, [Ca^2+^] transients remained unchanged from their unstretched values both before and after stunning. Stunning resulted in depressed contractile function in the CON group; however, the STM group exhibited no significant change in contractile function compared to before stunning.

**Figure 3:**
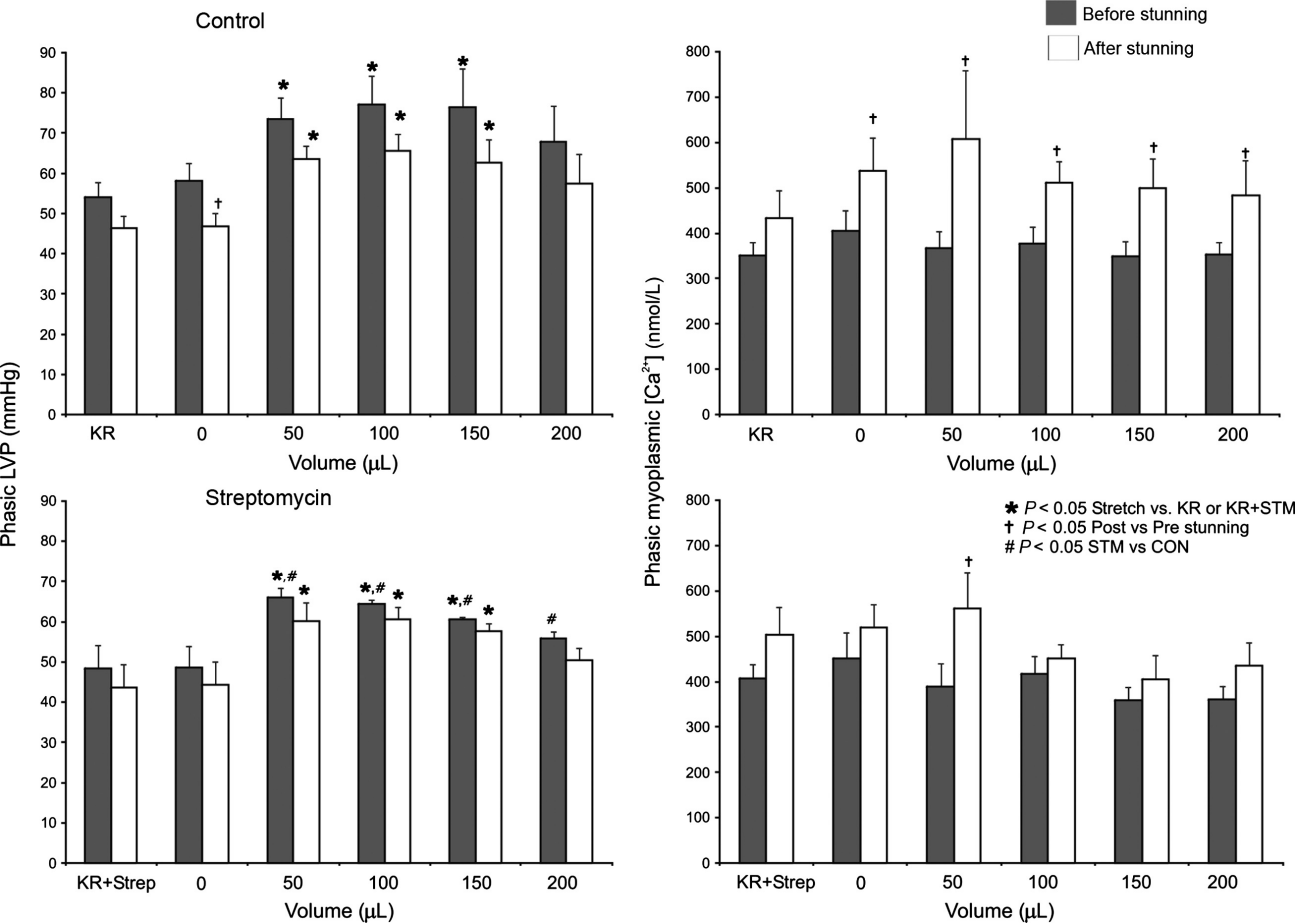
Summary of phasic (systolic-diastolic) LVP (left panel) and phasic [Ca^2+^] (right panel) at each stage of myocardial stretch before and after stunning in CON and STM groups. Phasic LVP increased with incremental stretch but was compromised at maximal stretch (200 μL) both before and after Stunning. The increases in phasic LVP with stretch (100–200 μL; *P* < 0.05) were reduced in the STM group versus the CON group before but not after stunning. Phasic [Ca^2+^] with stretch was higher after stunning in both groups but there was no difference between the CON and STM groups. Hearts in both groups exhibited similar trends in phasic LVP and phasic [Ca^2+^] with stretch after stunning but [Ca^2+^] was lower in the STM group with stretch to 100 and 150 μ/L (*P* < 0.05).

Summarized data of phasic (i.e., systolic-diastolic) LVP and [Ca^2+^] for CON and STM groups before and after stunning (Fig.3) showed no difference in phasic LVP and [Ca^2+^] between CON and STM hearts at baseline (KR or KR+Strep). Systematic stretch caused an increase in phasic LVP in CON and STM hearts both before and after stunning. Also, the increase in phasic LVP by stretch in the CON group was greater than that in the STM group before stunning. But no concurrent increase in phasic [Ca^2+^] was observed in either group by stretch before stunning. After stunning, phasic LVP in the CON hearts was decreased. However, there was no significant difference in phasic LVP before or after stunning in the STM treated hearts.

**Figure 4:**
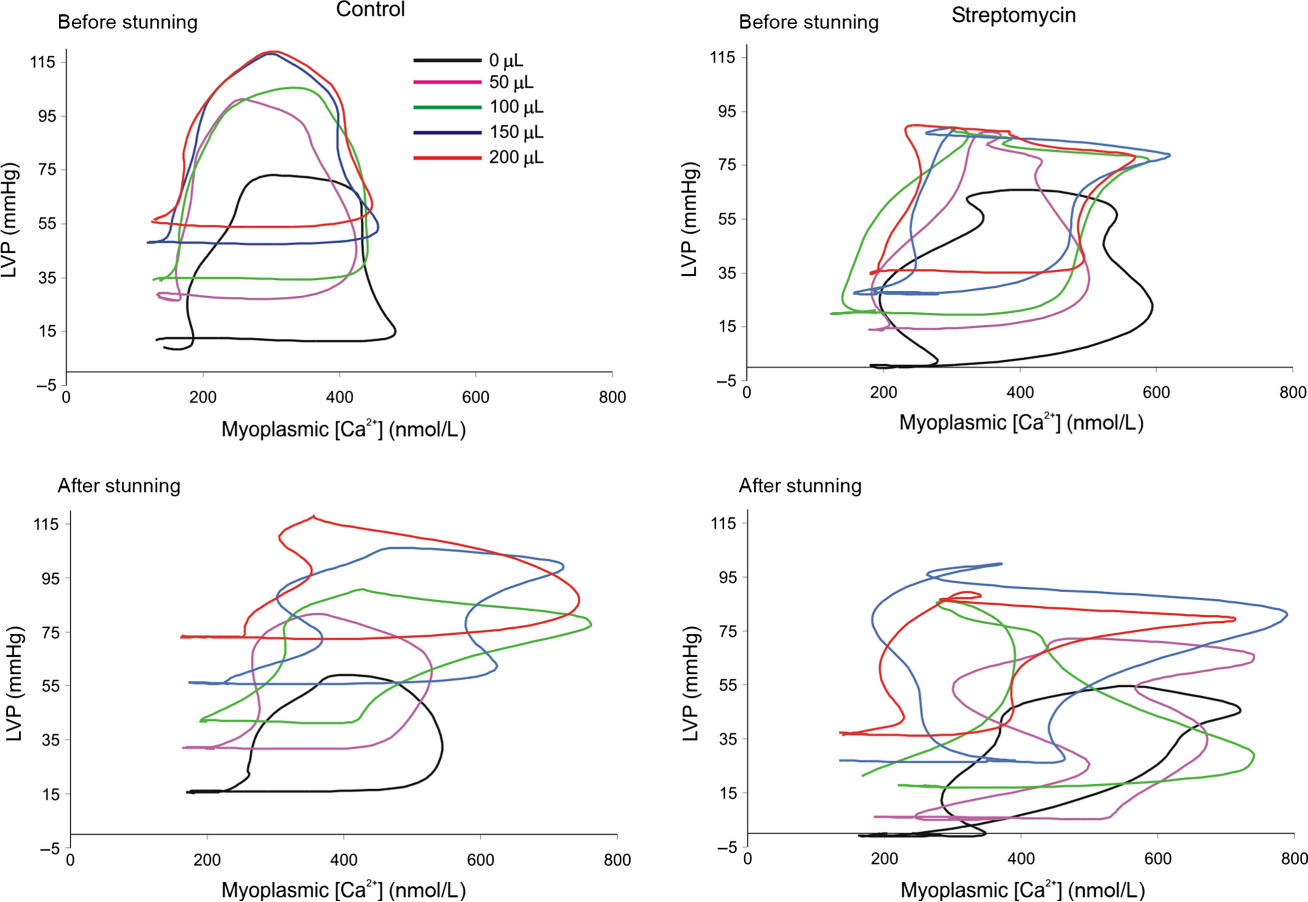
Representative phase–space diagrams of LVP versus [Ca^2+^] before and after stunning in STM-treated and CON hearts. These diagrams were quantified using indices of area (LVP × Ca^2+^) and orientation (phasic LVP/phasic [Ca^2+^]). Note the more uniform response to stretch before stunning than after stunning and the tendency of stretch to increase LVP but not [Ca^2+^] before stunning more so in the CON than in the STM group. After stunning, stretch increased both LVP and [Ca^2+^] in the CON group whereas LVP was reduced and [Ca^2+^] unchanged by stretch in the STM group.

Representative LVP · [Ca^2+^] loops for representative cardiac beats at increasing stretch before and after stunning in CON and STM hearts are shown (Fig.4). Note that CON hearts showed a predominantly classic pattern of an increase in [Ca^2+^] that preceded the increase in LVP before and after stunning. In contrast, STM treated hearts demonstrated predominantly an increase in LVP that occured simultaneously with an increase in [Ca^2+^] before and after stunning. The LVP · [Ca^2+^] area relationship became distorted after stunning as evidenced by the changes in the shapes of the loops. Note that after stunning phasic Ca^2+^ was elevated more so in CON than in STM un-stretched hearts.

**Figure 5:**
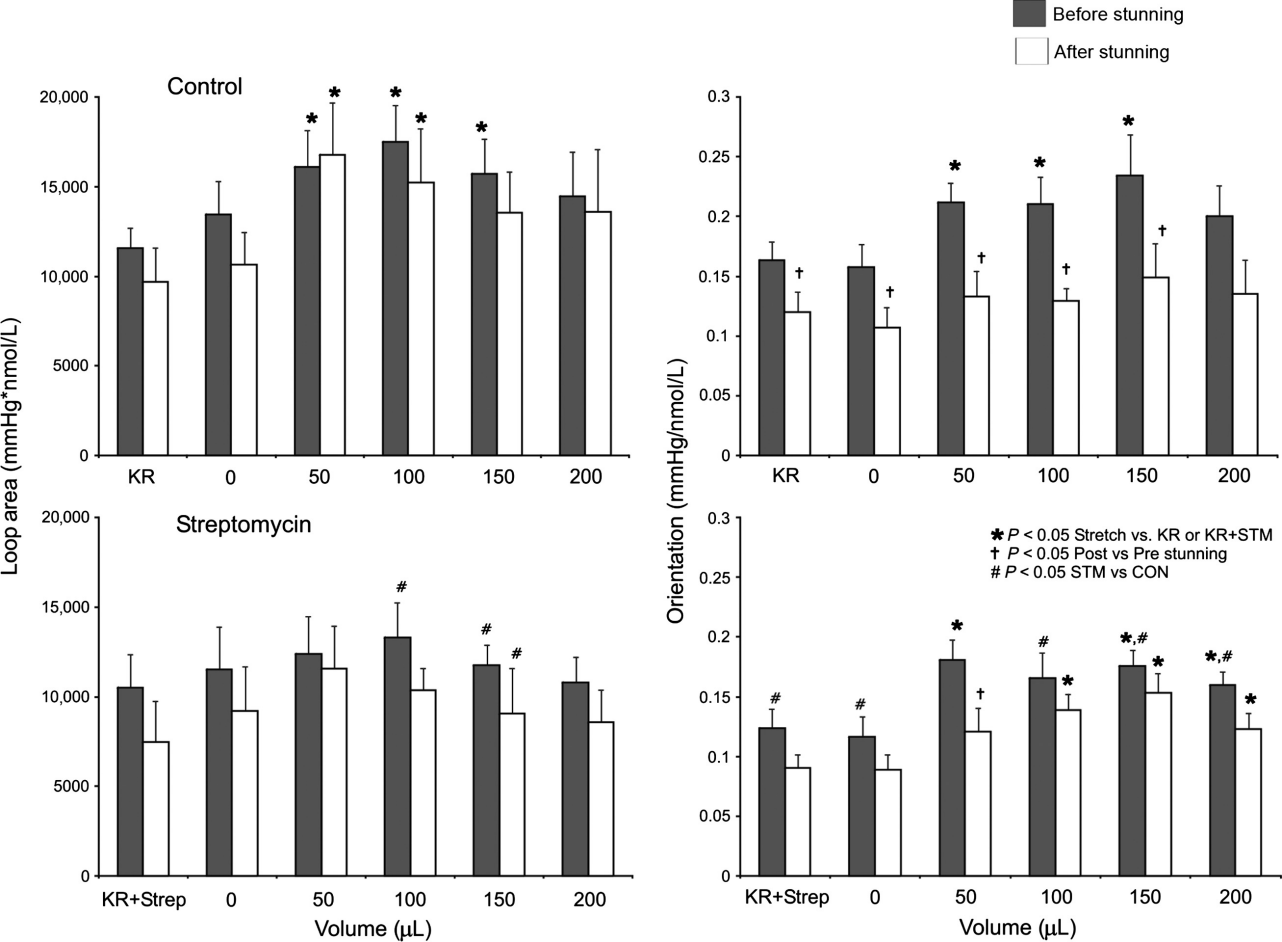
Summary of area (left panel) and orientation (right panel) of phase–space diagrams at each stretch before and after stunning in CON and STM groups. Loop area and orientation increased with stretch (50–150 μL) before stunning in CON hearts. Note that after stunning CON hearts displayed stretch-related increases in loop area, but not orientation, suggesting decreased efficiency of Ca^2+^-contraction coupling. STM-treated hearts did not exhibit increases in loop area with stretch before or after stunning. However, this group did exhibit stretch-related increases in orientation after stunning that suggested a better preservation of Ca^2+^-contraction coupling.

Summarized data for LVP · [Ca^2+^] area and LVP/[Ca^2+^] loop orientation (systolic − diastolic LVP/systolic − diastolic [Ca^2+^]), which characterize the changes in loop shape (Fig.5), showed that loop area increased mostly during stretch in CON hearts (primarily due to increased phasic LVP) but not in STM hearts. Loop orientation shifted upward in both CON and STM hearts with stretch before stunning, whereas with stretch after stunning, loop orientation tilted rightward in CON hearts and rightward and upward in STM hearts with stretch.

**Figure 6:**
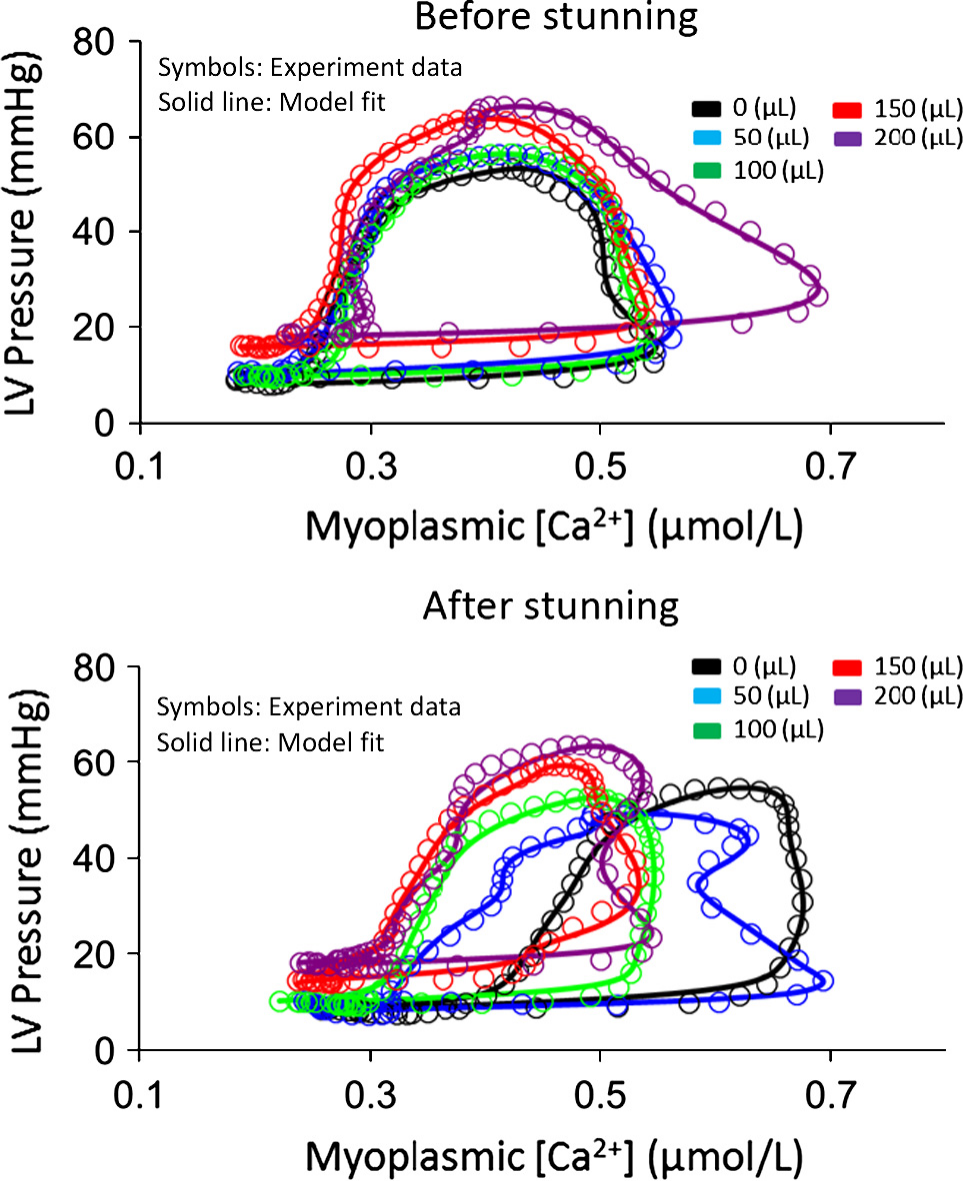
Phase–space characterization of experimental LVP (symbols) and modeled LVP (smooth line) versus myoplasmic [Ca^2+^] during myocardial stretch before and after stunning for one representative cycle in each stretch group. The model fitted the data with less than 5% error in any cycle.

Dynamic change in LVP as a function of the change in [Ca^2+^] are displayed during a cardiac cycle obtained from each stretch group before and after stunning (Fig.6). The model (smooth lines) closely fitted the experimental observations (circles).

### Excitation-contraction coupling modeling applied to myocardial stretch-activated channels

**Figure 7:**
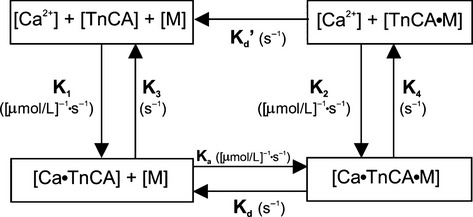
Block diagram of a biochemical model relating the input/output relationship between myoplasmic [Ca^2+^] and LVP adapted from Baran et al. (1997) and Shimizu et al. (2002) The four-state model is governed by five differential equations. TnCA represents the troponin C molecule on the actin (A) myofilament, M represents the myosin head, + indicates weak bonds and • represents strong bonds. The sequence of events from phasic [Ca^2+^] to contraction are as follows: Ca^2+^ binds to TnCA, tropomyosin shifts so M and A can bind forming an actinomyosin cross-bridge, Ca^2+^ dissociates from TnCA with cross-bridge attached, and finally the cross-bridge breaks. Note that A and M cannot form cross-bridges in the absence of Ca^2+^; however, since this is a loose coupling model, once a cross-bridge has been formed it no longer requires associated Ca^2+^ to remain attached. Adapted from Rhodes et al. (2006a,b). Model rate constants and their units are indicated by their sites of action and described in Table1. Model parameter values for stretch before and after stunning are given in Table2.

Table2 presents estimated changes in model parameters with stretch before and after stunning from the mathematical model used to interpret the kinetic and static data (Fig.7). Before stunning, stretching the myocardium suggested: (1) an increase in dynamic A·M cross-bridge formation (*α*_a_), but not in the interaction between TnCA and Ca^2+^ (*α*_1_); (2) a decrease in the resting (static) value of affinity for cross-bridge attachment (*β*_a_); (3) no change in the interaction between TnCA and Ca^2+^ (*β*_1_), and (4) no changes in the association rate constant of Ca^2+^ to A·M (*K*_2_), or in the dissociation rate constants of Ca^2+^ from TnCA (*K*_3_), of Ca^2+^ from A·M (*K*_4_), or of A·M with attached Ca^2+^ (*K*_d_) or unattached Ca^2+^ (*K*_d_′).

Stunning itself (BL 0 μL) suggested: (1) decreases in dynamic A·M cross-bridge formation (*α*_a_) and in the dynamic interaction between TnCA and Ca^2+^ (*α*_1_); (2) a large decrease in the resting (static) value of affinity for cross-bridge attachment (*β*_a_); (3) an increase in the resting interaction between TnCA and Ca^2+^ (*β*_1_), (4) decreases in the association rate constant of Ca^2+^ to A·M (*K*_2_) and in the dissociation rate constants of Ca^2+^ from TnCA (*K*_3_), (5) an increase in the dissociation rate constant of Ca^2+^ from A·M (*K*_4_), and (6) decreases in the dissociation rate constants of A·M with attached Ca^2+^ (*K*_d_) and with unattached Ca^2+^ (*K*_d_′).

After stunning, stretching the myocardium suggested: (1) an added increase in dynamic actinomyosin cross-bridge formation (*α*_a_) and a marked decrease in the dynamic interaction between TnCA and Ca^2+^ (*α*_1_); (2) a further marked decrease in the resting (static) value of affinity for cross-bridge attachment (*β*_a_) but no change in the resting interaction between TnCA and Ca^2+^ (*β*_1_), (3) marked decreases in the association rate constant of Ca^2+^ to A·M (*K*_2_) and in the dissociation rate constants of Ca^2+^ from TnCA (*K*_3_), (4) an increase of Ca^2+^ from A·M (*K*_4_), and decreases in the dissociation rate constants of A·M with attached Ca^2+^ (*K*_d_) and with unattached Ca^2+^ (*K*_d_′).

## Discussion

Stretch-activated (gated) ion channels (SAIC) are mechanotransducers that conduct ionic currents only when a stretch stimulus occurs in the cell membrane (Sukharev and Sachs 2012). Stretch or pressure causes the stretch-activated channels to undergo a conformational change that allows ions to enter the cell. The probability of opening decreases when the stimulus is removed. The ions conducted are generally nonspecific and their effect is to depolarize the cell membrane. Stretch (increased volume) of the myocardial wall is believed to enhance myocardial contractility. A superfamily of SAICs is the transient receptor potential (TRP) channel (Patel et al. 2010; Yin and Kuebler 2010). Although TRP channels are activated by ligands, voltage, and temperature, they are also involved in mediating mechano-transduction. The TRPC6 channel is expressed in the cardiovascular system and is a sensor of mechanical-induced membrane stretch (Patel et al. 2010). A newly characterized signaling cascade in cardiac and skeletal muscle is a mechanotransduction element in the microtubular network that activates Nox2-dependent ROS (X-ROS) generation during mechanical stretch (Prosser et al. 2013a,b; Ward et al. 2014).

The purpose of our study was to understand the effect of endogenous stretch-induced cation channels on changing the relationship between beat-to-beat phasic myoplasmic [Ca^2+^] and cardiac contractility (phasic LVP) before and after short ischemic stunning. We blocked these channels with STM and used a mathematical model of Ca^2+^ handling and actinomyosin cross-bridge cycling to aid in assessing the mechanism of stretch-induced changes in contractility and myoplasmic Ca^2+^ transients. Overall, our results demonstrate that: (1) Increasing balloon volume to stretch the LV is a valid model of enhanced contractility. (2) Stretch-induced enhanced contractility is not a result of increased myoplasmic [Ca^2+^]. (3) Improved return of contractile function after stunning when stretch activated channels are blocked may be mediated by improved Ca^2+^ handling and/or increased actinomyosin cross-bridge cycling (enhanced sensitivity). (4) Cardioprotection imparted by STM may also be related to attenuated Ca^2+^ loading on reperfusion after ischemic stunning. The cardioprotective effects of STM are also noted in the change in loop orientation (horizontal vs. vertical dimension, Fig.4). CON hearts show changes in loop orientation after stunning that occur when the increase in [Ca^2+^] is not accompanied by a concomitant increase in contraction. On the other hand, the STM hearts demonstrate changes in loop orientation after stunning that suggest that the increase in [Ca^2+^] is accompanied by an increase in contraction. Therefore STM blunted, but did not completely block, the stretch-induced increase in phasic LVP compared to the CON group (Fig.3). This was also evident in the loop area (Fig.5), which was greater in the CON versus STM group with stretch. However, there was no significant difference in phasic LVP before or after stunning in the STM hearts. This suggests that STM has cardioprotective effects after ischemia because cardiac damage due to stunning may be in part mediated by stretch activated-cation channels.

In a prior report (Rhodes et al. 2006a) we found that a similar four-state model with cooperativity was capable of interpreting changes in central and downstream regulation of contractility in the presence and absence of IR injury in isolated hearts that was based on the phase-–space relationship between [Ca^2+^] and LVP. We reported that IR injury resulted in reduced Ca^2+^ affinity for TnCA and decreased cross-bridge kinetics. In this report we examined for changes in Ca^2+^-contraction coupling due to stretch in normal and stunned cardiac muscle. It is evident that stretch does not affect this relationship via the upstream mechanism since there is no change in [Ca^2+^] with stretch either before or after stunning, although stunning itself does can result in an increase in phasic [Ca^2+^]. Consequently, we focused on the changes in central (Ca^2+^ affinity for TnCA) and downstream (A·M cross-bridge interaction) using the model.

Before and after stunning, stretch resulted in increased sensitivity of TnCA for Ca^2+^, although this effect was blunted in the stunned myocardium, as is noted from the lower values of *α*_1_ and *K*_3_ with stunning. Stretch also increased cooperativity for attachment of actinomyosin cross bridges before and after stunning. However, stunned myocardium also showed a concomitant decrease in cross-bridge dissociation rates that may directly relate to a decrease in available ATP due to stunning or fewer functioning cross-bridges. The stretch-related decrease in cross-bridge dissociation during moderate stretch exhibited by stunned myocardium may be a compensatory mechanism by functioning myocytes to improve contractile function. When the effects of stretch on normal versus stunned myocardium are compared, it is evident that at each stretch level both the cooperative interaction between Ca^2+^ and TnCA, and the cooperative formation of actinomyosin cross-bridges, are reduced in the stunned tissue. Stunning also results in depressed myofilament Ca^2+^ sensitivity in the presence of attached cross-bridges, regardless of stretch.

It appears that in the nonstunned heart, myofilament Ca^2+^ sensitivity is depressed while cross-bridge association is enhanced dramatically in the early stages of stretch (Table2). Closer to maximal stretch myofilament Ca^2+^ sensitivity returns to the unstretched baseline values and cross-bridge association remains high, but plateaued. This suggests that the first mechanism responsible for increased contractility during stretch is an increase in the formation of cross-bridges whereas the central mechanism responds as stretch increases.

There are several proposals for cooperative mechanisms of cross-bridge formation (Moss et al. 2004; Rice and de Tombe 2004). To give one example, in a Markov model of cardiac thin filament activation (Campbell et al. 2010), simulations of the cooperativity in both steady-state and dynamic force–Ca^2+^ relationships indicated that the interaction of two adjacent tropomyosins (Tm–Tm) in a regulatory unit composed of actin (A) monomers, TnC, TnI (inhibitory subunit) and Tm, was sufficient to explain cooperativity. The model analysis suggested that Tm–Tm coupling potentiates the activating effects of strongly bound cross-bridges and contributes to force–Ca^2+^ dynamics in intact cardiac muscle.

Our interpretation of the stretch-receptor–myoplasmic [Ca^2+^] relationship with stunning, aided by this mathematical model of cross-bridge cooperativity, is as follows: (1) Stunning (IR) results in greater myoplasmic [Ca^2+^] but reduced Ca^2+^ affinity in the unstretched myocardium. However, stretch itself does not result in significant changes in measured myoplasmic [Ca^2+^] before or after stunning. (2) Cooperativity in cross-bridge kinetics and myofilament Ca^2+^ handling is depressed after as compared to before stunning in the unstretched myocardium. This may be related to fewer functioning cross-bridges. (3) Stunning results in depressed myofilament Ca^2+^ sensitivity in the presence of attached cross-bridges regardless of stretch as interpreted from the model parameters in Table2. In the nonstunned heart myofilament Ca^2+^ sensitivity is depressed while cross-bridge association increases dramatically in the early stages of stretch. (4) Cross-bridge dissociation rates *K*_d_ and *K*_d_′ are largely depressed after stunning which might be related to decreased ATP synthesis. The stretch-related decrease in cross-bridge dissociation during moderate stretch exhibited by stunned myocardium may be a compensatory mechanism by functioning myocytes to improve contractile function.

### Potential limitations

This model of Ca^2+^-contraction coupling relied on Ca^2+^ transients obtained via an optic probe with a transmural measurement field and surface area of approximately 3 mm^2^, under which the detected signal diminished proportional to depth. The model parameters were optimized to best-fit isovolumic LVP, a more global measurement. One of the implicit assumptions of this model was that the local Ca^2+^ transient is a true representation of Ca^2+^ kinetics in the entire ventricular wall regardless of orientation of the individual myocytes, which are known to be markedly different from endocardium to epicardium (Campbell et al. 2005). The use of a thin intra-cavitary balloon to increase volume to stretch the myocardium may not accurately mimic the condition of overall greater heart volume to stretch the heart. Further studies using greater stretching and higher doses of STM may help to further elucidate the role of Ca^2+^ in stretch-induced increases in cardiac contraction. However, higher concentrations of STM (IC_50_ of 1–2 mmol/L) also inhibited myogenic tone and K^+^-induced isometric force largely by blocking L-type, dihydropyridine-sensitive Ca^2+^ channels (Miller and Langton 1998).

## References

[b1] Aldakkak M, Camara AK, Heisner JS, Yang M, Stowe DF (2011). Ranolazine reduces Ca^2+^ overload and oxidative stress and improves mitochondrial integrity to protect against ischemia reperfusion injury in isolated hearts. Pharmacol. Res.

[b2] An J, Varadarajan SG, Novalija E, Stowe DF (2001). Ischemic and anesthetic preconditioning reduces cytosolic [Ca^2+^] and improves Ca^2+^ responses in intact hearts. Am. J. Physiol. Heart Circ. Physiol.

[b3] Baran D, Ogino K, Stennett R, Schnellbacher M, Zwas D, Morgan JP (1997). Interrelating of ventricular pressure and intracellular calcium in intact hearts. Am. J. Physiol.

[b4] Brandes R, Figueredo VM, Camacho SA, Baker AJ, Weiner MW (1993a). Investigation of factors affecting fluorometric quantitation of cytosolic [Ca^2+^] in perfused hearts. Biophys. J.

[b5] Brandes R, Figueredo VM, Camacho SA, Baker AJ, Weiner MW (1993b). Quantitation of cytosolic [Ca^2+^] in whole perfused rat hearts using Indo-1 fluorometry. Biophys. J.

[b6] Burkhoff D (1994). Explaining load dependence of ventricular contractile properties with a model of excitation-contraction coupling. J. Mol. Cell. Cardiol.

[b7] Bustamante JO, Ruknudin A, Sachs F (1991). Stretch-activated channels in heart cells: relevance to cardiac hypertrophy. J. Cardiovasc. Pharmacol.

[b8] Calaghan SC, White E (1999). The role of calcium in the response of cardiac muscle to stretch. Prog. Biophys. Mol. Biol.

[b9] Calaghan SC, Belus A, White E (2003). Do stretch-induced changes in intracellular calcium modify the electrical activity of cardiac muscle?. Prog. Biophys. Mol. Biol.

[b10] Campbell K (1997). Rate constant of muscle force redevelopment reflects cooperative activation as well as cross-bridge kinetics. Biophys. J.

[b11] Campbell KB, Wu Y, Simpson AM, Kirkpatrick RD, Shroff SG, Granzier HL (2005). Dynamic myocardial contractile parameters from left ventricular pressure-volume measurements. Am. J. Physiol. Heart Circ. Physiol.

[b12] Campbell SG, Lionetti FV, Campbell KS, McCulloch AD (2010). Coupling of adjacent tropomyosins enhances cross-bridge-mediated cooperative activation in a markov model of the cardiac thin filament. Biophys. J.

[b13] Chen Q, Camara AK, An J, Novalija E, Riess ML, Stowe DF (2002). Sevoflurane preconditioning before moderate hypothermic ischemia protects against cytosolic [Ca^2+^] loading and myocardial damage in part via mitochondrial K_ATP_ channels. Anesthesiology.

[b14] Eisenberg E, Hill TL (1985). Muscle contraction and free energy transduction in biological systems. Science.

[b15] Grynkiewicz G, Poenie M, Tsien RY (1985). A new generation of Ca^2+^ indicators with greatly improved fluorescence properties. J. Biol. Chem.

[b16] Hill TL (1983). Two elementary models for the regulation of skeletal muscle contraction by calcium. Biophys. J.

[b17] Kentish JC, Wrzosek A (1998). Changes in force and cytosolic Ca^2+^ concentration after length changes in isolated rat ventricular trabeculae. J. Physiol.

[b18] Landesberg A, Sideman S (1994). Mechanical regulation of cardiac muscle by coupling calcium kinetics with cross-bridge cycling: a dynamic model. Am. J. Physiol.

[b19] von Lewinski D, Stumme B, Maier LS, Luers C, Bers DM, Pieske B (2003). Stretch-dependent slow force response in isolated rabbit myocardium is Na+ dependent. Cardiovasc. Res.

[b20] Miller AL, Langton PD (1998). Streptomycin inhibition of myogenic tone, K^+^-induced force and block of L-type calcium current in rat cerebral arteries. J. Physiol.

[b21] Moss RL, Razumova M, Fitzsimons DP (2004). Myosin crossbridge activation of cardiac thin filaments: implications for myocardial function in health and disease. Circ. Res.

[b22] Patel A, Sharif-Naeini R, Folgering JR, Bichet D, Duprat F, Honore E (2010). Canonical TRP channels and mechanotransduction: from physiology to disease states. Pflugers Arch.

[b23] Peterson JN, Hunter WC, Berman MR (1991). Estimated time course of Ca^2+^ bound to troponin C during relaxation in isolated cardiac muscle. Am. J. Physiol.

[b24] Prosser BL, Khairallah RJ, Ziman AP, Ward CW, Lederer WJ (2013a). X-ROS signaling in the heart and skeletal muscle: stretch-dependent local ROS regulates [Ca^2+^]_i_. J. Mol. Cell. Cardiol.

[b25] Prosser BL, Ward CW, Lederer WJ (2013b). X-ROS signalling is enhanced and graded by cyclic cardiomyocyte stretch. Cardiovasc. Res.

[b26] Rhodes SS, Ropella KM, Audi SH, Camara AK, Kevin LG, Pagel PS (2003a). Cross-bridge kinetics modeled from myoplasmic [Ca^2+^] and LV pressure at 17° C and after 37° C and 17° C ischemia. Am. J. Physiol. Heart Circ. Physiol.

[b27] Rhodes SS, Ropella KM, Camara AK, Chen Q, Riess ML, Stowe DF (2003b). How inotropic drugs alter dynamic and static indices of cyclic myoplasmic [Ca^2+^] to contractility relationships in intact hearts. J. Cardiovasc. Pharmacol.

[b28] Rhodes SS, Camara AK, Ropella KM, Audi SH, Riess ML, Pagel PS (2006a). Ischemia reperfusion dysfunction changes model-estimated kinetics of myofilament interaction due to inotropic drugs in isolated hearts. Biomed. Eng. Online.

[b29] Rhodes SS, Ropella KM, Camara AK, Chen Q, Riess ML, Pagel PS (2006b). Ischemia-reperfusion injury changes the dynamics of Ca^2+^-contraction coupling due to inotropic drugs in isolated hearts. J. Appl. Physiol.

[b30] Rice JJ, de Tombe PP (2004). Approaches to modeling crossbridges and calcium-dependent activation in cardiac muscle. Prog. Biophys. Mol. Biol.

[b31] Rice JJ, Jafri MS, Winslow RL (1999a). Modeling gain and gradedness of Ca^2+^ release in the functional unit of the cardiac diadic space. Biophys. J.

[b32] Rice JJ, Winslow RL, Hunter WC (1999b). Comparison of putative cooperative mechanisms in cardiac muscle: length dependence and dynamic responses. Am. J. Physiol.

[b33] Shimizu J, Todaka K, Burkhoff D (2002). Load dependence of ventricular performance explained by model of calcium-myofilament interactions. Am. J. Physiol. Heart Circ. Physiol.

[b34] Stowe DF, Varadarajan SG, An J, Smart SC (2000). Reduced cytosolic Ca^2+^ loading and improved cardiac function after cardioplegic cold storage of guinea pig isolated hearts. Circulation.

[b35] Sukharev S, Sachs F (2012). Molecular force transduction by ion channels: diversity and unifying principles. J. Cell Sci.

[b36] Varadarajan SG, An J, Novalija E, Smart SC, Stowe DF (2001). Changes in [Na^+^]_i_, compartmental [Ca^2+^], and NADH with dysfunction after global ischemia in intact hearts. Am. J. Physiol. Heart Circ. Physiol.

[b37] Ward ML, Williams IA, Chu Y, Cooper PJ, Ju YK, Allen DG (2008). Stretch-activated channels in the heart: contributions to length-dependence and to cardiomyopathy. Prog. Biophys. Mol. Biol.

[b38] Ward CW, Prosser BL, Lederer WJ (2014). Mechanical stretch-induced activation of ROS/RNS signaling in striated muscle. Antioxid. Redox Signal.

[b39] Yeung EW, Whitehead NP, Suchyna TM, Gottlieb PA, Sachs F, Allen DG (2005). Effects of stretch-activated channel blockers on [Ca^2+^]_i_ and muscle damage in the mdx mouse. J. Physiol.

[b40] Yin J, Kuebler WM (2010). Mechanotransduction by TRP channels: general concepts and specific role in the vasculature. Cell Biochem. Biophys.

